# *Citrobacter koseri* infection of the nose masquerading as keratoacanthoma: A case report and review of the literature

**DOI:** 10.1016/j.jdcr.2025.09.035

**Published:** 2025-10-10

**Authors:** Grace Sora Ahn, Allison M. Han, Zhe Jessie Hou, Brian Hinds, Shang I. Brian Jiang

**Affiliations:** Department of Dermatology, University of California San Diego, La Jolla, California

**Keywords:** Citrobacter koseri, iatrogenic infection, keratoacanthoma, pseudocarcinomatous hyperplasia, SSTI

## Introduction

Citrobacter koseri (C. koseri), formerly known as C. diversus, is a rare Gram-negative bacillus and known colonizer of the gastrointestinal tract that infrequently causes infections. Though an uncommon agent of disease, C. koseri can cause meningitis and central nervous system abscesses in neonates and immunocompromised individuals when pathogenic.^1^ Urinary tract infections and respiratory infections from C. koseri may occasionally be observed in hospitalized patients with multiple comorbidities.^1^ Skin and soft tissue infections (SSTI) due to C. koseri; however, are exceedingly rare, with only 4 out of 1562 reported cases of SSTIs demonstrating growth of this bacteria in an Antimicrobial Surveillance Program.^2^ Literature on C. koseri SSTIs is limited to a handful of case reports describing folliculitis, cellulitis, and abscesses.^3^, ^4^, ^5^, ^6^, ^7^, ^8^, ^9^ Herein, we present a unique case of pseudocarcinomatous hyperplasia secondary to C. koseri on the nose mimicking eruptive keratoacanthoma clinically and histopathologically. To our knowledge, this is the first case of a C. koseri tunneling infection inducing cutaneous neoplasia-like changes.

## Case

**Fig 1:**
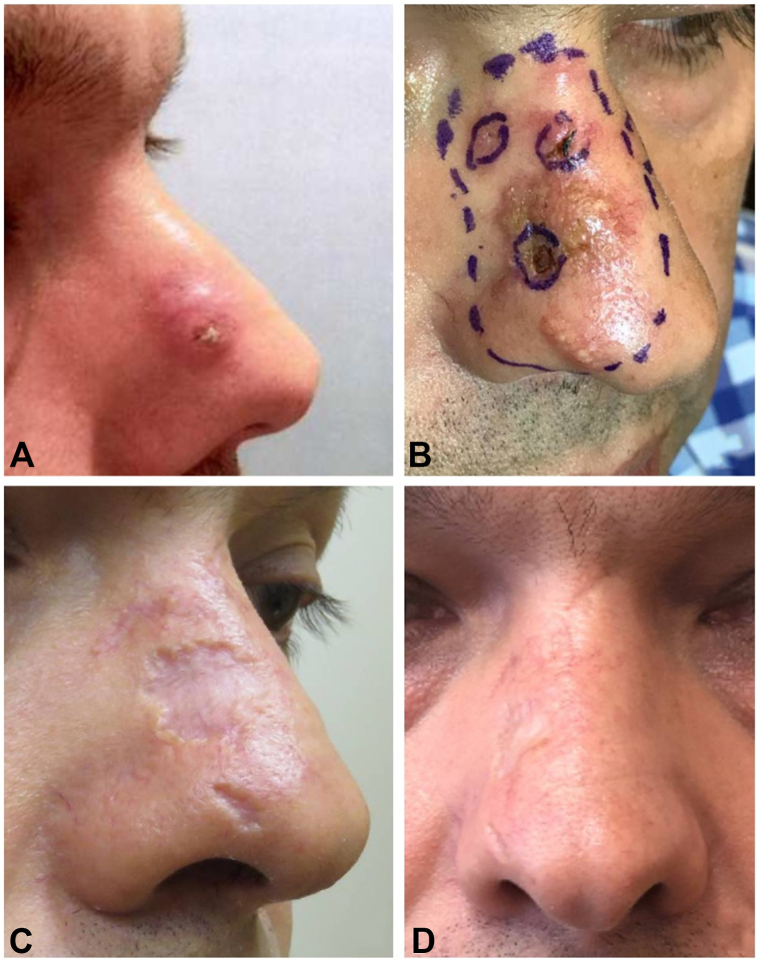
Initial onset with acneiform papule of the right nasal sidewall **(A)**; erythematous scaly plaque after 2 months at presentation **(B)**; mildly atrophic residual scars postantibiotic treatment **(C)**; lack of recurrence after 3 years **(D)**.

A 40 year-old man presented to our dermatologic surgery center for multiple growing lesions on the right nasal sidewall. The lesion initially presented as a single lesion 4 months prior (Fig 1, *A*) and had undergone 3 biopsies suggesting invasive squamous cell carcinoma with a well-differentiated keratoacanthoma-like growth pattern. Following a negative CT scan of the head and neck, surgery and radiation were recommended. The patient presented for a second opinion after additional consultation with 2 plastic surgeons, by which time, the lesion had grown into a 3-cm plaque with 4 satellite lesions.

**Fig 2:**
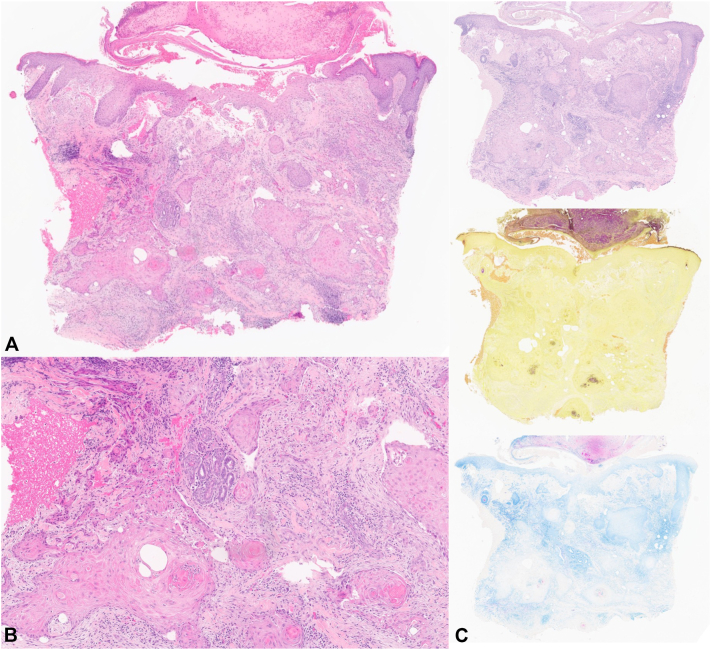
Keratoacanthoma-like endophytic metaplastic proliferation of the right nasal sidewall **(A)**. Close-up of interconnecting cyst-like spaces in background of syringosquamous metaplasia and numerous neutrophilic microabscesses along adnexal structures **(B)**. PAS-D (*top right*), Gram (*middle right*), AFB (*bottom right*) stains were all negative **(C)**.

Physical examination revealed several erythematous crusted plaques and papules on the right nasal sidewall, extending from the proximal bridge to the distal tip (Fig 1, *B*). Three scouting punch biopsies were performed which revealed endophytic keratinocytic proliferations with interconnecting cyst-like spaces, syringosquamous metaplasia, and numerous neutrophilic microabscesses (Fig 2, *A* and *B*). While fungal and mycobacterial cultures as well as PAS, AFB, gram stains were negative (Fig 2, *C*), tissue culture was notable for C. koseri growth confirming the diagnosis of a multifocal KA-like hyperplastic response to infection.

Upon further history, the patient reported that the nasal lesions developed after a recent sophagogastroduodenoscopy procedure and progressed into the large plaque with satellite nodules as observed at presentation. Given the timing, the lesions are suspected to be a result of iatrogenic inoculation of the nasal skin with C. koseri, a common gastrointestinal microbe, likely during the withdrawal of the sophagogastroduodenoscopy tubing. Rapid resolution following a 14-day course of ciprofloxacin allowed the patient to avoid surgery and radiation which was initially recommended for the patient. The lesions healed with mildly atrophic scars at 2-month and eventual 1-year follow-up (Fig 1, *C*). No evidence of recurrence was noted during follow-up over a 3-year period (Fig 1, *D*).

## Discussion

**Fig 3:**
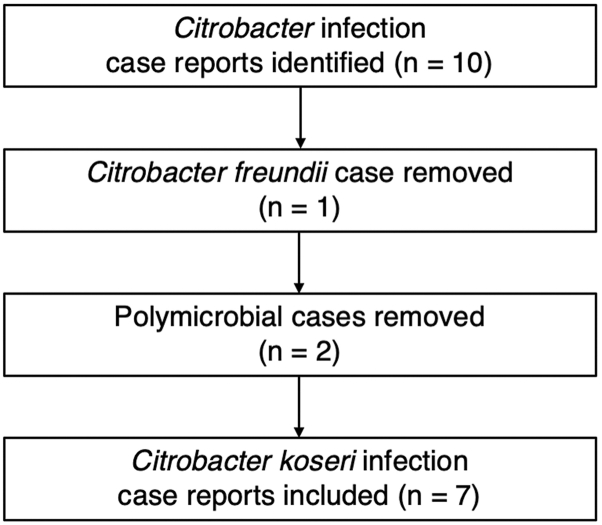
Systematic PubMed/MEDLINE literature review of Citrobacter skin and soft tissue infection case reports.

A comprehensive review was performed using PubMed/MEDLINE to identify C. koseri-related SSTIs comprising terms such as “Citrobacter koseri,” “Citrobacter diversus,” “skin infection” and related variations. Only 10 cases of Citrobacter SSTIs have been reported in the literature, 2 of which were polymicrobial, 1 of which was found to be Citrobacter freundii (Fig 3). With the exception of 1 abscess, all cases resolved completely with antibiotic therapy alone, without the need for procedural intervention (Table I).

**Table I:** Summary of reported cases of Citrobacter koseri skin and soft tissue infections

Infection type	Age/Sex	Pathogen	Comorbidity	Source	Surgery	Therapy	Reference
Cellulitis	30 M	Citrobacter diversus (koseri)	MM	Community	none		Bishara et al 1998
Folliculitis	28 M	Citrobacter diversus (koseri)	Acne	Community	none	Trimethoprim-sulfamethoxazole	Chastain et al 2000
Scalp folliculitis	49 M	Citrobacter koseri	Folliculitis decalvans + rosacea	Community	none	Ciprofloxacin	Garcia-Bustinduy et al 2002
Cellulitis	60 F	Citrobacter koseri	B-cell CLL	Community	none	Amoxicillin-clavulanic acid and ofloxacin	Kluger et al 2010
Facial nodules	86 M	Citrobacter koseri	None	Community	none	Amoxicillin/Clavulanic acid	Sherif et al 2018
Facial folliculitis	15 M	Citrobacter koseri	Acne	Community	none	Ceftriaxone	Raia et al 2015
Axillary cellulitis + abscess	16 F	Citrobacter koseri	Acne	Community	I&D	Ciprofloxacin and clindamycin	Khreis et al 2025
KA-like syringosquamous metaplasia	40 M	Citrobacter koseri	None	Nosocomial	none	Ciprofloxacin	Current case

*BC-CLL*, B-cell-chronic lymphocytic leukemia; *F*, female; *KA*, keratoacanthoma; *M*, Male; *MM*, multiple myeloma.

Pseudoepitheliomatous hyperplasia is a benign reactive process often associated with infections that can mimic keratoacanthoma and squamous cell carcinoma histologically. Typically presenting as a well-demarcated crusted nodule or plaque, pseudoepitheliomatous hyperplasia—also referred as pseudocarcinomatous hyperplasia—may arise from various causes, with deep fungal or mycobacterial infections being the most commonly implicated. While C. koseri has been associated with colonic hyperplasia mimicking gastrointestinal adenomas and carcinomas, infections have not previously been known to masquerade as cutaneous carcinomas.^10^

Clinicians, including dermatologists, should remain aware of the uncommon but critical possibility that infections from rare pathogens can produce marked hyperplastic changes that mimic carcinoma histologically. This case also underscores the critical role of clinical vigilance, emphasizing the need for thorough history-taking and infectious workup—including tissue cultures—when the clinical course does not align with the presumptive histopathologic diagnosis. With timely diagnosis and comprehensive evaluation, patients may avoid potentially harmful surgical or radiation interventions and instead achieve resolution with appropriate antibiotic therapy alone.

## Conflicts of interest

None disclosed.
